# Human‐modified habitats change patterns of population genetic structure and group relatedness in Peter's tent‐roosting bats

**DOI:** 10.1002/ece3.2255

**Published:** 2016-07-29

**Authors:** Maria Sagot, Caleb D. Phillips, Robert J. Baker, Richard D. Stevens

**Affiliations:** ^1^ Department of Biological SciencesState University of New York at OswegoOswegoNew York13126; ^2^ Department of Biological SciencesTexas Tech UniversityLubbockTexas79409; ^3^ Department of Natural Resources ManagementTexas Tech UniversityLubbockTexas79409

**Keywords:** Habitat scales, human‐modified habitats, relatedness, roosts, tent‐roosting bats

## Abstract

Although coloniality is widespread among mammals, it is still not clear what factors influence composition of social groups. As animals need to adapt to multiple habitat and environmental conditions throughout their range, variation in group composition should be influenced by adaptive adjustment to different ecological factors. Relevant to anthropogenic disturbance, increased habitat modification by humans can alter species’ presence, density, and population structure. Therefore, it is important to understand the consequences of changes to landscape composition, in particular how habitat modification affects social structure of group‐forming organisms. Here, we combine information on roosting associations with genetic structure of Peter's tent‐roosting bats, *Uroderma bilobatum* to address how different habitat characteristics at different scales affect structure of social groups. By dividing analyses by age and sex, we determined that genetic structure was greater for adult females than adult males or offspring. Habitat variables explained 80% of the variation in group relatedness (mainly influenced by female relatedness) with roost characteristics contributing the most explained variation. This suggests that females using roosts of specific characteristics exhibit higher relatedness and seem to be philopatric. These females mate with more males than do more labile female groups. Results describe ecological and microevolutionary processes, which affect relatedness and social structure; findings are highly relevant to species distributions in both natural and human‐modified environments.

## Introduction

Social organization is one of the most important features in animal societies that responds not only ecological, but also social selective pressures (Ross and Keller 1995). As group formation has important fitness implications (e.g*.,* protection from predators and thermoregulation ability), understanding mechanisms whereby individuals form stable groups has interested scientists for more than a century (Galton 1871). However, many unanswered questions remain (Krause and Ruxton 2002). Multiple studies have suggested that animal associations and cohesiveness are enhanced by: (1) limited and patchily distributed resources (e.g*.,* Altmann 1974); (2) female recruitment into natal groups followed by long‐term philopatry (Wilkinson 1985; Kerth et al. 2000; Castella et al. 2001); (3) or high degrees of relatedness within groups (Ross 2001). Nonetheless, high group stability has been found in places where resources are plentiful (Rossiter et al. 2002) and in groups with female and/or male natal dispersal (reviewed in Clutton‐Brock 1989).

Ecological factors such as distribution and patchiness of suitable habitats and resources, fragmentation, and changing environmental conditions (Christiansen and Reyer 2011; Zachos and Hartl 2011) are also known to be important determinants of social structure (i.e., group formation, size, composition, stability), as they alter the costs–benefits of social interactions (Bronikowski and Altmann 1996; Pusey & Packer 1997). Thus, variation in social structure should be expected among and within populations as a consequence of differences in adaptive adjustment of males and females to differences in the ecological environment (Rubenstein 1980; Dunbar 1981; Campbell 2008; Chaverri and Kunz 2010). Although social groups vary in the strength of social interactions (e.g., Sterck 1998; Grassi 2006; Moore et al. 2008) as they adapt to different habitat and environmental conditions, how these factors interrelate to shape social structure has escaped the focus of contemporary research. Here, we combine information on habitat selection at multiple scales, roosting associations, and population genetic structure, to address how different habitat characteristics at different scales affect structure of social groups.

Studies on the effect of habitat use, especially in resource‐defense polygyny mating systems, have traditionally been evaluated at only one scale, even though it is known that animals use the habitat differently at different scales (e.g., Morris 1987). Moreover, although it is known that the rapid rate and extent of habitat modification by humans influence genetic structure, rate of population differentiation and extinction among others (Saunders et al. 1991; Couvet 2002; Dodd and Kashan 2003; Manel et al. 2003; Allendorf and Luikart 2007; Bloor et al. 2008; Lawton‐Rauh 2008; Walker et al. 2008; Mayer et al. 2009), social structure, and ecology of urban species remain poorly understood (Shochat et al. 2006). Increased understanding of species that inhabit human‐modified areas will comprise a significant component to understanding the future of global biodiversity (Chace and Walsh 2006).

Peter's tent‐roosting bat, *Uroderma bilobatum,* is an ideal species to test habitat (both natural and human‐modified) effects on social structure. *Uroderma bilobatum* is a fruit‐eating bat found in tall‐standing tropical forests from Oaxaca and Veracruz, Mexico, south to Peru, Bolivia, and southeastern Brazil (Davis 1968), at elevations ranging from 0 to 1800 m (Davis 1968). *Uroderma bilobatum* roosts in tents constructed from large leaves of various species of plants, modified by cutting veins and leaflets to form a semi‐enclosed space (Kunz and Lumsden 2003). *Uroderma bilobatum* mating system is defined as a resource‐defense polygyny, in which males defend roosts to monopolize access to females (Kunz & McCraken 1996). Thus, social groups consist of one male, multiple females, and their dependent young (Baker and Clark 1987; LaVal and Rodríguez‐Herrera 2002). Solitary males are usually found in roosts that are in close proximity to social groups, presumably to try to get access to females (M. Sagot, pers. obs.). To avoid inbreeding in resource‐defense polygyny mating systems, males, females, or both commonly disperse from the natal habitat as they approach maturity (Greenwood 1980). Although *U. bilobatum* can use multiple native plants as tents, Sagot et al. (2013) found that the species is more abundant in human‐altered habitats and prefers to roost in introduced coconut palms. Higher density of bats is found in coconut palms 8–15 m tall, with tents that range from 5 to 10 m in height (Sagot et al. 2013). This suggests that human activity is facilitating use of non‐natural habitats, which may in turn influence population structure and patterns of relatedness within and among groups.

Here, we aim to: (1) describe genetic structure of the Peter's tent‐roosting bats at a local and regional scale; (2) determine relative contributions of different habitat (both natural and human‐modified) factors on group relatedness and distribution of group genetic structure at three different kinds of environmental levels: roosts (variables describing roosts characteristics), structural (habitat characteristics within habitat patches), and macrohabitat (e.g*.,* land use categories, geographical variables); and (3) elucidate patterns and mechanisms of female/male natal dispersal and long‐term associations.

Our study is the first in associating the use of habitat and limited resources at multiple scales with resource‐defense polygyny mating systems. Moreover, we add a novel component, which is how the introduction of non‐native resources, such as coconut palms, changes habitat selection preferences and affects social structure. Our methods provide a promising approach to understanding the ecological and microevolutionary processes shaping genetic structure in wild populations.

## Methods

### Study site and sampling

**Figure 1:**
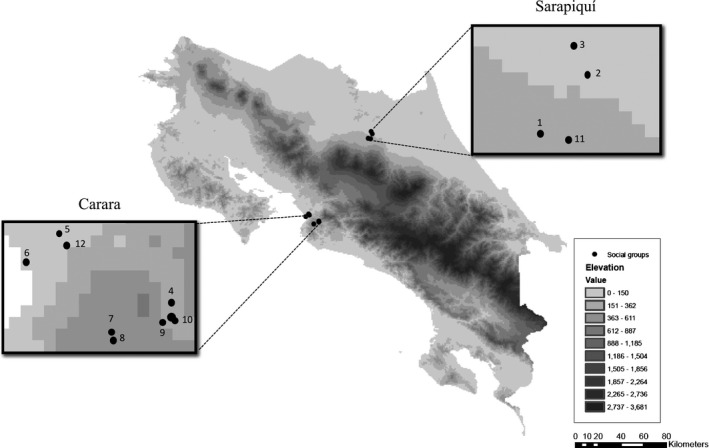
A map of Costa Rica depicting the study regions and the location of 12 social groups.

Fieldwork was conducted at Carara National Park and surrounding areas (9°44′55.78″ N, 84°37′1.29″ W) in the Central Pacific versant and in the region of Sarapiquí (10°23′55.88″ N, 84°08′06.23″’ W) on the Caribbean versant of Costa Rica between June 2007 and May 2009 (Fig. 1). Sarapiquí, Heredia province, is considered a plain; however, it also has a mountainous topography due to close proximity to the Central Volcanic Mountain Chain (Sanford et al. 1984). Land use encompasses grasses, forests, reforestation lands, seasonal and annual plantations (e.g., banana, coffee, and pineapple), and urban populations. Sarapiquí has a tropical climate with a dry season (extending from March to May) and a rainy season (from May to February; Sanford et al. 1984). The average annual temperature is 26–28°C, and the humidity ranges from 80% to 90% annually. (Sanford et al. 1984). The second site, Carara, belongs to Puntarenas province. This region encompasses three life zones: tropical humid forest with transition to per‐humid, super‐humid tropical forest with transition to humid, and super‐humid premontane forest with transition to basal per‐humid (Boza and Cevo 1998). Primary forest occupies most of the area, surrounded by isolated urban populations and farmland (Boza and Cevo 1998). The Central Volcanic Cordillera, which is an important biogeographic barrier in Costa Rica, separates both regions (Janzen 1983).

These two regions were selected for the study because of their differences in human settlement history and presence of bats, and native and introduced plants (used by bats for roost construction). A previous study by Sagot et al. (2013), investigating habitat effects on presence and density of bats, determined that *U. bilobatum* was not found in the forests of Sarapiquí; however, it was abundant in human‐modified areas, where it roosts in coconut palms and banana plants. Coconut palms (*Cocos nucifera*) are native to coastal areas of South‐East Asia (Malaysia, Indonesia, Philippines; Chan and Elevitch 2006). They were introduced into West Africa and the Caribbean (including Atlantic and Pacific coast of Central America) during the 16th century by European explorers (Harries 1978). Currently, coconut palms have a wide pantropical distribution (Chan and Elevitch 2006). Their natural habitat is the narrow sandy coast, but their local distribution has expanded due to human introductions as agricultural plantations or ornamentals. In the Sarapiquí region, native plants used as roosts (e.g., *Atthalea* spp.) are not present or in low abundance (Grayum 2003). Moreover, the first coconut palm and banana plantations in Sarapiquí are reported around the year 1961 (Joyce 2006). Before this time, most of the region was composed by primary forests (Joyce 2006). This suggests that *U. billobatum* was not present or in very low abundance in the region before the introduction of these two plant species. On the other hand, in the Carara region, the native *Atthalea* spp. and *Cryosophila* spp. palms (both used by *U. billobatum* for roost construction) are found in forested areas (Grayum 2003); suggesting that *U. bilobatum* was found in this region before introduction of coconut palms. Still, they have adapted to use these introduced plants, and currently, they are more abundant in human‐modified habitats than in the forest (Sagot et al. 2013).

A detailed description of the sampling methodology at both regions, including the time span of data collection, can be found in Sagot et al. (2013). Briefly, to find *U. bilobatum* in Sarapiquí and Carara, we visited all plant species known to be used as roosts (Kunz and Lumsden 2003; Rodríguez‐Herrera et al. 2007) in forested and human‐modified habitats, covering approximately the same area in both types of habitat (area determined on a georeferenced map in ArcGIS 9.3.2; ESRI 2009). In forests, we followed available trails and we created two 10 Km transects per site, by placing them randomly over a georeferenced study area map, using ArcGIS. Human‐modified areas were defined as plantations, grassland, or urbanized sites. In these areas, we followed available streets and roads, covering approximately the same distance covered in the forest.

For this study, we selected four different sites in Sarapiquí out of the total sampling area. The closest sites in this region were 3 Km apart. The largest distance between two sites was 15 Km. In the Carara region, we selected five different sites out of the total sampling area. The closest distance between two sites was 4.6 Km, and the largest distance was 13 Km. The closest distance between two sites among regions (Sarapiquí and Carara) was 70.2 Km, and the largest distance was 99.4 Km. For every group found, we recorded the Global Positioning System (GPS) coordinates, and we captured the entire group using a hand net with an extendible pole. From each bat, we recorded age, sex, weight, forearm measurement, and reproductive status. For the study, we used 187 individuals of 588 captured total. We vouchered 30 individuals (specimens and tissues [heart, liver, lung, kidney, muscle, and embryo], and we deposited them at the Genetic Resources Collection, Natural Science Research Laboratory, Texas Tech University museum). We released the rest of the individuals on site after collecting two 5‐mm wing punches. We recorded macrohabitat, microhabitat, and structural characteristic variables from the occupied tents. We defined macrohabitat as discrete habitat types in the landscape based on land use (human‐modified/forest), site (Carara vs. Sarapiquí), and distance to the forest (measured from the georeferenced map using ArcGIS). We set to 0 the distance from the forest for roosts found in the forest. We defined microhabitat as particular habitat subsets within a macrohabitat measured in a 20‐m‐diameter plot around the roost‐containing plant. The variables that we measured were as follows: amount of herbaceous cover, number of bushes (woody plants with a diameter at breast height (DBH) < 20 cm), number of trees (woody plants with a DBH larger than 20 cm), average tree diameter at breast height (DBH), and average light penetration measured with a quantum light meter (Hydrofarm West, model 2053; Hydrofarm, Petaluma, CA) taken at cardinal points. Structural characteristics reflected attributes of roosts. The variables we measured were as follows: tent height and plant height (measured using a Suunto PM5/66PC clinometer; Vantaa, Finland), and plant species (represented by dummy variables in analyses; Suits 1957).

### Sequencing and genotyping

We found and extracted DNA from 187 *U. bilobatum* (96 adult females, 13 adult males, and 78 offspring, Table 1) belonging to 12 social groups. We made the extractions from liver, kidney, or wing punch tissues preserved in lysis buffer. We isolated DNA by either organic protocols (Longmire et al. 1997) or using a Qiagen DNeasy Blood and Tissue Kit (Qiagen Inc., Chatsworth, CA).

**Table 1:** Number of males, females, offspring per social group, and group FIS at each of the studied regions

Group	Number of adult males	Number of adult females	Number of Offspring	Group FIS	Region
1	1	12	11	0.34	Sarapiquí
2	1	4	4	0.25	Sarapiquí
3	1	10	9	0.29	Sarapiquí
4	1	24	24	0.28	Carara
5	1	10	10	0.31	Carara
6	1	5	4	0.38	Carara
7	2	6	3	0.23	Carara
8	1	6	3	0.22	Carara
9	1	6	5	0.42	Carara
10	1	4	0	0.44	Carara
11	1	4	0	0.51	Sarapiquí
12	1	5	5	0.19	Carara

We amplified the entire *cyt‐b* gene (1140 bases) using LGL765, LGL766 primer combination (Cathy et al. 1998) with the following thermal profile: 94°C for 3:30 min, 34 cycles of 94°C for 30 sec, 57°C for 30 sec, 72°C for 1:15 min, and a final extension at 72°C for 3 min. PCR products were purified using QIAquick PCR Purification Kit (Qiagen Inc.). Sequencing reactions used a set of internal sequencing primers: Uro_cytb_seq_F (5’‐CGG CTT CTC CGT AGA CAA AG‐3’) and Uro_cytb_seq_R (5’‐TGG GAT ACC TGT TGG GTT GT‐3’) and Big Dye version 3.1 (Applied Biosystems, Foster City, CA) with the following thermal profile: 94°C for 4 min, 34 cycles at 94°C for 30 sec, 57°C for 30 sec, 60°C for 4 min. Sequences were resolved using an ABI PRISM 3100‐Avant (Applied Biosystems), and verified and aligned using Sequencher version 4.9 (Gene Code Corporation, Ann Arbor, MI).

We amplified ten microsatellite loci previously developed by Sagot et al. (2014), following the protocol outlined in that study. Loci were fluorescently labeled following the M13 protocol developed by Schuelke (2000). We size resolved loci using an ABI PRISM 3100‐Avant (Applied Biosystems), and genotype calls were determined using GeneMapper version 4.0 (Applied Biosystems).

### Genetic structure at multiple levels

We used Collapse v1.2 (http://darwin.uvigo.es) to determine the distribution and frequency of mtDNA haplotypes, and we constructed a maximum parsimony haplotype network using TCS v1.21 (http://darwin.uvigo.es; Templeton et al. 1992; Clement et al. 2000). We mapped distributions of mtDNA haplotypes among social groups onto this network.

To characterize group genetic structure and population differentiation, we calculated pairwise *F*_ST_ values from the microsatellite and *cyt‐b* data matrixes, respectively, using ARLEQUIN v3.1 (Excoffier et al. 2005) at group (pairwise *F*_ST_ among the twelve social groups), locality (pairwise *F*_ST_ comparing different localities within Sarapiquí and Carara), and regional (pairwise *F*_ST_ comparing Sarapiquí and Carara) levels. Significance was assessed using 10,000 permutations (sequential Bonferroni method implemented in ARLEQUIN).

To evaluate whether levels of differentiation among social groups were greater than that expected by chance, for the mitochondrial data we randomly drew 15 haplotypes (equal to the average group size) and 11 haplotypes (based on the mode) from the haplotype frequency table through 100 iterations. We next calculated pairwise *F*_ST_ values among replicates, and compared the observed distribution of *F*_ST_ *P*‐values to the permuted distribution of *F*_ST_ *P*‐values using a Mann–Whitney *U*‐test. We repeated the same procedure for microsatellite data, but in this case, we randomly drew 15 and 11 individuals through 100 iterations. We also compared the proportion of significant pairwise *F*_ST_ *P*‐values between the *cyt‐b* and microsatellite datasets using a *Z*‐test.

To determine whether patterns of population differentiation are associated with geographic distance between social groups, we performed a mantel test using IBD Web Service v3.21 (IBDW 3.21; Jensen et al. 2005), with significance determined through 30,000 permutations. Fixation index (FST) Genetic (mtDNA) and geographic (measured in ArcGIS) distance matrices were log‐transformed prior to analysis as suggested by Slatkin (1993) and Hutchison and Templeton (1999).

### Spatial genetic structure

To evaluate spatial genetic structuring, we used the Bayesian model‐based clustering method implemented in STRUCTURE (Pritchard et al. 2000), which assigns individuals to populations based on multilocus genotypes. For *K* population clusters, we estimated the probability of the data and individual membership to each cluster using a Markov chain Monte Carlo method (MCMC). We run the program assuming independent allele frequencies and admixture (Pritchard et al. 2000). We conducted three independent runs for each value of *K* to determine the most likely number of clusters, implementing 100,000 iterations after a burn‐in period of 1,000,000 iterations. We determined the number of populations best fitting the data using the log probability Pr(*X*|*K*) and Δ*K*, as described by Evanno et al. (2005) and implemented in the program STRUCTURE HARVESTER (Dent and vonHoldt 2011). Simulated values of *K* ranged from 1 to 12 reflecting the number of social groups in the study. We also combined social groups into seven localities (4 in Carara and 3 in Sarapiquí; simulated *K* ranged from 1 to 7) to test for locality level structure. We then combined localities into regions (Carara and Sarapiquí; simulated *K* ranged from 1 to 2) to assess regional level structure. Subsequent analyses assessed population structure separately for adult males, adult females, and offspring. This was conducted to explore whether optimal *K* when combining all sexes and ages was influenced by the structure of adult females, adult males or offspring within social groups. The simulated value of *K* for these analyses ranged from 1 to 12.

### Habitat effects on group structure

To determine relative contribution of structural, microhabitat, and macrohabitat scales on patterns of group relatedness, we performed variance partitioning analyses (Legendre and Legendre 1998) whereby group fixation index (*F*_IS_) was treated as the dependent variable and the different sets of habitat characteristics represented three independent explanatory variables. We conducted this test to characterize: (1) unique variation explained by a particular set (i.e., macrohabitat, microhabitat, or structural) after controlling for the other two sets, (2) correlated variation explained by each two‐way interaction, (3) correlated variation explained by all explanatory variables, and (4) variation not accounted for by any explanatory variable. We conducted the tests in VarCan (version 1, Peres‐Neto et al. 2006). For sets contributing significantly to the variation in *F*_IS_, we conducted multiple regression analyses between *F*_IS_ and variables within a given set, controlling for group size, to determine specific variables that contributed more to the explained variance. W performed these tests in R v2.10 (R Development Core Team 2009). Assumptions of the tests were tested before analyses.

### Relatedness and mating patterns

As groups are formed by one or two adult males, multiple adult females, and their offspring, overall patterns of group structure might be influenced by adult females and/or of offspring within social groups. If patterns were influenced by offspring, we expected at least in some groups that harem males sired all, or most offspring and thus, high offspring *F*_IS_ within these groups. Moreover, an offspring‐only variance partitioning analysis should resemble the above‐described variance partitioning analysis (habitat variables should explain the offspring *F*_IS_ variance in a similar manner). On the other hand, if the *F*_IS_ among adult females was the primary driver of the overall pattern, we expected that habitat variables explain female *F*_IS_ variance in a similar manner as the overall variance partitioning analysis. To determine genetic signal for these predictions, we performed variance decomposition analyses for adult females and offspring separately based on *F*_IS_ and sets of environmental characteristics as explained above. We calculated group, adult female, and offspring *F*_IS_ in ARLEQUIN v3.1 (Excoffier et al. 2005).

To determine male monopolization and extra‐pair/extra‐group mating patterns, we performed a paternity analysis. We calculated paternity assignment using a maximum‐likelihood‐based method described in Marshall et al. (1998) and implemented in the program CERVUS v3.0 (Kalinowski et al. 2007). CERVUS assigns to each offspring tested the most likely candidate parent with a predetermined level of confidence. We considered the harem males at each region as potential fathers for each offspring. Although we assigned mothers to all embryos, we were unable to identify the mother of the offspring that were born, as they detached from the mother's nipple during capture. We calculated the error rate by randomly resampling 20 individuals.

Because it was not possible to sample all males in our study sites, to estimate the number of males siring offspring within sampled social groups, we identified paternal half‐siblings (offspring that share the same father) among pups roosting in the same tent. To do this, we used the likelihood‐based method implemented in the program KINSHIP v1.3 (Goodnight and Queller 1999). Using allele frequencies generated in CERVUS, KINSHIP performs maximum‐likelihood tests of pedigree relationships between pairs of individuals by calculating likelihood ratios when comparing a hypothesis of relatedness for all possible individual pairs in the dataset to a null hypothesis of no relatedness.

We calculated the log‐likelihood ratio that shared alleles are identical by paternal descent (Rp = 0.50, Rm = 0.00), and compared ratios to a null hypothesis that alleles are not identical by descent through either route of Mendelian transmission (Rp = 0.00, Rm = 0.00). We then performed 1000 pairs of simulation routines to generate a probability of the likelihood ratio for each pair. Offspring were identified as paternal half‐siblings if the *P*‐value for that dyad was smaller than 0.05.

To examine the effects of male tenure in female dispersal within social groups, we performed a simple regression to compare adult female and offspring relatedness (calculated using the software MLRelate [Kalinowski et al. 2006]). This test was performed in R v2.10 (R Development Core Team 2009). Individuals with multiple genotypes missing were eliminated from the analyses. We performed these analyses for the 10 social groups that had offspring.

## Results

Raw diversity of microsatellite dataset was described by a mean of 9.4 alleles per locus. Mean expected heterozygosity and mean polymorphic loci were 0.61 and 0.58, respectively. All loci used in the study were in Hardy–Weinberg equilibrium. We found 49 unique *cyt‐b* haplotypes, distributed among 12 social groups (Figure S1). The most common haplotype was present in 28 individuals distributed among seven social groups. Two other common haplotypes were identified, occurring in 23 and 17 individuals, which were distributed among 6 and 5 social groups, respectively. The remaining haplotypes occurred at low frequencies and were sometimes found in more than one social group, but often only occurring in a single group.

### Genetic structure at multiple levels

**Figure 2:**
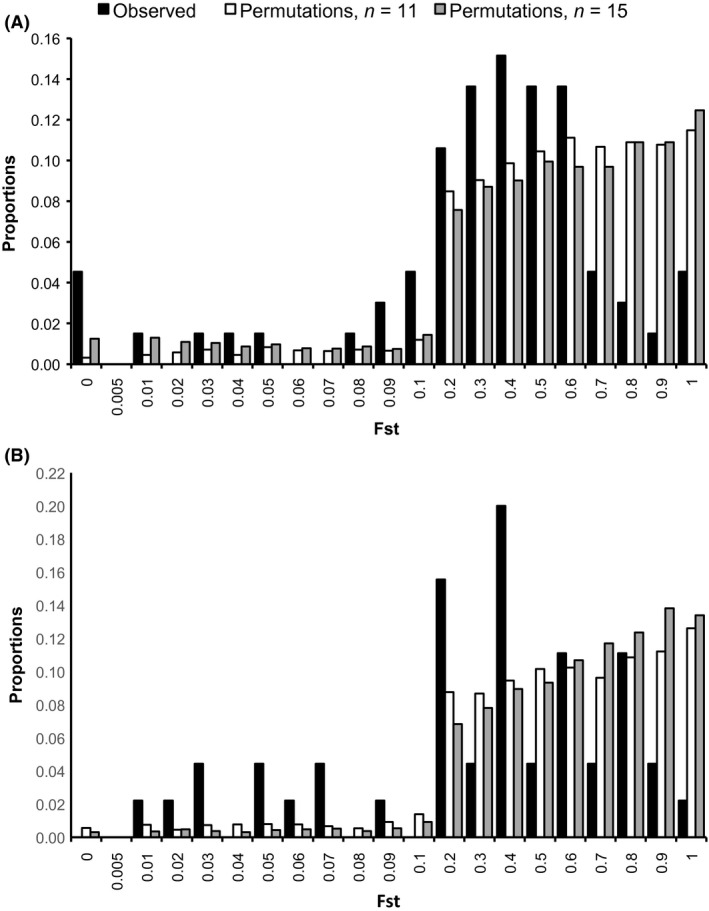
Proportion of pairwise *F*_ST_ values for observed and permutated (A) mitochondrial haplotypes and (B) microsatellite loci.

Pairwise *F*_ST_ among social groups estimated from the mitochondrial dataset revealed that within Sarapiquí region, only two social groups were significantly different from each other. However, within Carara region, social groups were more differentiated. At a regional level, Sarapiquí and Carara were not significantly different (*F*_ST_ = 0.0033, *P *=* *0.4355). Comparing the distribution of observed *F*_ST_ *P*‐values using the *cyt‐b* gene among groups to the randomly permuted distributions indicated that the observed frequency distribution of *F*_ST_ *P*‐values was significantly smaller (i.e., more significant *P*‐values) than that expected by chance when the group size was 11 (observed = 0.353 ± 0.242, permuted = 0.532 ± 0.278; *U* = 103172, *P* < 0.001), as well as when the group size was 15 (observed = 0.353 ± 0.242, permuted = 0.522 ± 0.295; *U* = 108871, *P* < 0.001) (Fig. 2A). Pairwise *F*_ST_ estimated from the microsatellite dataset at the regional level revealed that Sarapiquí and Carara were not significantly different (*F*_ST_ = 0.0055, *P *=* *0.2754). Comparison of the distribution of observed *F*_ST_ values among groups to the randomly permuted distributions indicated again that the observed frequency distribution of *F*_ST_ *P*‐values was significantly smaller than that expected by chance when group size was 11 (observed = 0.370 ± 0.279, permuted = 0.533 ± 0.286; *U* = 75410, *P* < 0.001) and when group size was 15 (observed = 0.370 ± 0.279, permuted = 0.577 ± 0.272; *U* = 65992, *P* < 0.001) (Fig. 2B). In addition, the proportion of significant *F*_ST_ values for the *cyt‐b* and the microsatellite datasets were not significantly different (*Z* = 0.8, *P* = 0.448), indicating that signal for sex‐biased dispersal is not evident through comparison of marker types at this spatiotemporal scale. Observed patterns of group differentiation were not clearly associated with geographic distance; geographic distance between social groups did not explain significant variation in microsatellite genetic distance (IBD, Mantel test: *R *=* *0.143, *P *=* *0.143).

### Spatial genetic structure

Structure analyses revealed a maximum Pr(*X*|*K*) for *K* = 2 for all social groups, which corresponded to each of the regions (Sarapiquí and Carara). This pattern was consistent for females, males, and offspring. This pattern in contrast to the previous result of nonsignificant pairwise *F*_ST_ between regions indicated that regional differentiation is present, but weak. Within regions, Sarapiquí also showed a maximum Pr(*X*|*K*) for *K* = 2. On the other hand, there was substructure within Carara (Pr(*X*|*K*) for *K* = 5).

### Habitat effects on group genetic structure

**Figure 3:**
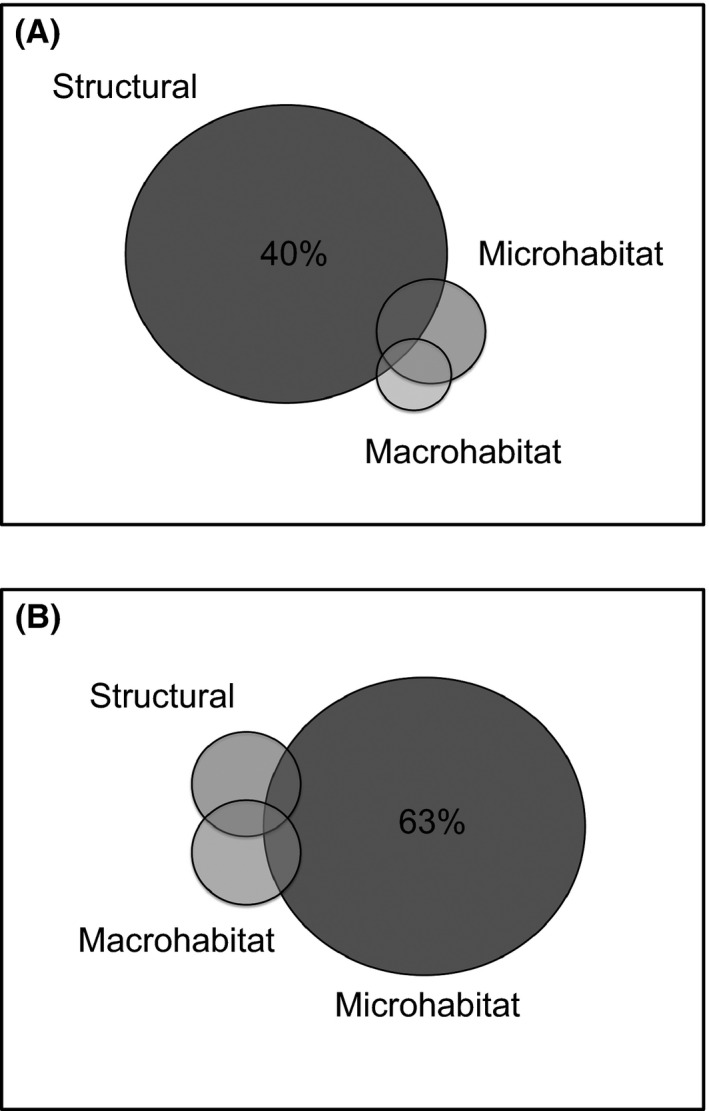
Variance partitioning analysis to determine structural, microhabitat, and macrohabitat effects on (A) group *F*_IS_ and (B) adult female *F*_IS_. Each box represents 100% of observed variation, with total area encompassed by the three habitat variables (three circles) representing the overall variance explained. Nonoverlapping areas represent unique variance explained by individual habitat variables. Overlapping areas indicate variance explained by the interaction of habitat variables. Nonsignificant variances are not reported.

To determine whether habitat could explain these patterns, we performed a variance decomposition with habitat variables and group *F*_IS_. We found that 80% of the variation in *F*_IS_ could be explained by habitat variables (*P *=* *0.001; Fig. 3A). From this explained variation, structural characteristics had the highest predictive power (40%) and this was the only scale that accounted for significant variation in *F*_IS_. Multiple regression between *F*_IS_ and variables from the structural scale demonstrated that groups with higher *F*_IS_ are found in coconut palms (*C. nucifera*) with heights that ranged from seven to 10 m, or in tents with heights that ranged from 10 to 15 m (*R* ^2^ = 0.4; *F*_4,10_ = 3.7; *P *=* *0.04).

### Group genetic structure and mating patterns

To investigate whether the overall distribution of genetic variance was driven by female *F*_IS_ within social groups, we performed a variance decomposition but using only the *F*_IS_ of adult females within social groups. We found that habitat variables explained 63% of variation (*P *=* *0.001, Fig. 3B). Females exhibited higher *F*_IS_ in habitats described by a small number of trees and low light abundance (*R* ^2^ = 0.6; *F*_5,6_ = 4.4; *P *=* *0.04). Offspring *F*_IS_ variation could not be attributed to any variable measured at any scale (*P *=* *0.211).

Moreover, because groups are also composed by multiple offspring, which can be sired by a single‐ or multiple‐related or nonrelated males, the pattern described above can also be influenced by offspring relatedness within social groups. Thus, we determined paternity and calculated the probability of sharing the same father. On a strict level (95% confidence), fathers were assigned to only 18 of 76 offspring (24%), and at a relaxed level (80% confidence), fathers were assigned to 39 offspring (51%). Subsequently, we found that within a given social group there were on average three males siring offspring (Table 2).

**Table 2:** Number of offspring per group and average number of males siring offspring per group

Group	Number of offspring	Number of fathers
1	11	3
2	4	1
3	9	6
4	24	7
5	10	4
6	4	4
7	3	3
8	3	3
9	5	3
10	4	3

**Figure 4:**
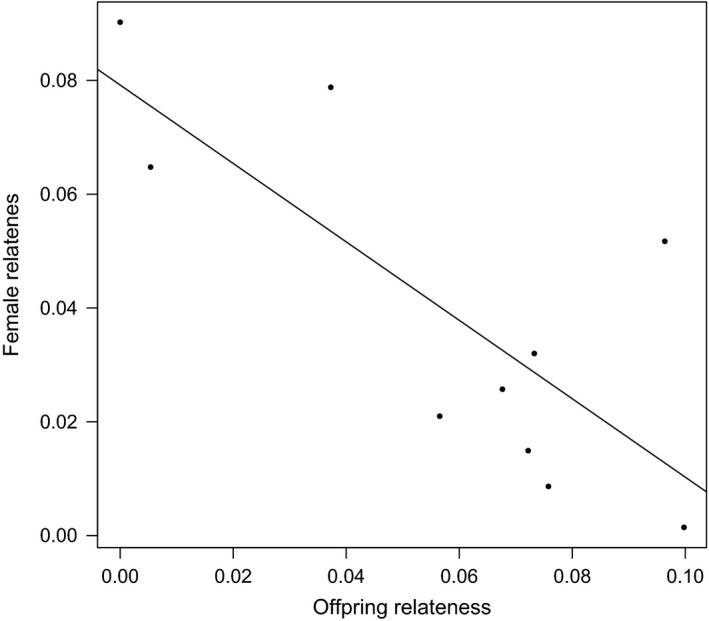
Regression of relatedness between adult female and offspring within social groups.

We found a negative and a highly significant relationship between adult female and offspring relatedness within groups (*R* ^2^ = 0.59; *F*_1,8_ = 11.71; *P *=* *0.009; Fig. 4); that is, for social groups in which adult females were more closely related to each other, offspring exhibited relatively lower relatedness values (i.e*.,* sired by multiple males). Conversely, groups consisting of less related adult females included relatively more related offspring (i.e*.,* sired by one or few males).

## Discussion

Analyses of genetic structure across multiple scales using mtDNA and nuclear microsatellite markers revealed that Peter's tent‐roosting bats exhibit significant structure among social groups within both regions (mainly within the Carara region), but not between regions (Sarapiquí vs. Carara). Within regions, patterns of genetic variation were not explained by geographic distance, but were attributed to habitat characteristics, indicating that genetic variation at local scales is shaped by social structure.

### Habitat effects on group genetic structure

Formation of cohesive groups has been reported in multiple bat species (e.g., Wilkinson 1985; Heckel et al. 1999; Kerth et al. 2000; Vonhof et al. 2004; Buchalski et al. 2014). Plausible hypotheses to explain these cohesive associations include knowledge of suitable foraging and roosting sites, thermoregulation, avoidance and reduced exposure to parasites and diseases, and cooperation (Allen 1962; Emlen 1994; Clutton‐Brock 2002; Kerth et al. 2002, 2008; Altizer et al. 2003; Calisher et al. 2006). It is also known that variation in relatedness among social groups could be a consequence of adaptation to different habitat conditions (Rubenstein 1980; Dunbar 1981; Chaverri and Kunz 2010). In this study, variation in group, and especially female genetic structure, appeared to be a consequence of preference for specific habitat characteristics, specifically the presence of coconut palms. Females roosting in coconut palms with specific characteristics (i.e*.,* heights ranging from 7 to 10 m) exhibited more structured social groups. It has been shown that adult females and daughters are able to use the same plant for multiple years (Lewis 1992), but when conditions become unsuitable, they move to establish their residence at a different site. Results indicating that social groups established in less preferred habitats (i.e*.,* plants other than coconut palms, in forested areas – Sagot et al. 2013) are less structured than in preferred habitats, suggesting that less preferred habitat characteristics promote female dispersal.

### Male tenure and female mating patterns

In addition to putative influences of roost suitability, female dispersal has also been coupled with resource‐defense polygyny (Greenwood 1980); the ability of males to defend a particular resource, such as refuges, for multiple mating seasons. If competition for resources is high and a proportion of males are prevented from breeding, males able to hold defendable resources (i.e*.,* roosts) have more chances of mating (Greenwood 1980). In these cases, the ability of males to defend a resource may promote female dispersal to avoid inbreeding or gain higher reproductive success, if male tenure exceeds female age at first conception (Clutton‐Brock 1989). Although no specific information is available for Peter's tent‐roosting bats, average age of maturity for phyllostomid bats is 4.6 months (Barclay and Harder 2003). Furthermore, a closely related species, Thomas's fruit‐eating bat, *Dermanura watsoni,* reaches sexual maturity at around 50 days after birth (Chaverri and Kunz 2006). Younger age at sexual maturity is advantageous in foliage roosting animals because the vulnerable roosting conditions favor offspring that attain flight and foraging independence faster (Chaverri and Kunz 2006). Therefore, it would be expected to find a similar developmental rate in Peter's tent‐roosting bats. Moreover, as males have been found using the same plant in multiple years (M. Sagot, unpubl. data), male tenure appears to be extensive in this species.

In this study, we found that offspring had lower relatedness when they belonged to social groups composed of females exhibiting higher relatedness, which can be due to multigenerational use of the same roosting site. On the other hand, higher relatedness was common among offspring in groups with less related females. As *U. bilobatum* male tenure likely exceeds female age at maturity, this pattern suggests that when females are philopatric (i.e., females belonging to one or few maternal lines due to multigenerational use of the same roosting site), they engage in extra‐pair/extra‐group mating (e.g*.,* mating with males other than the harem male) at a higher frequency, compared to females that disperse before attaining sexual maturity. Extra‐group paternity is especially likely in Peter's tent‐roosting bats due to the nature of their roosting ecology. Coconut palm roosts are highly clumped and remain usable across multiple mating seasons (Sagot and Stevens 2012; Sagot et al. 2013). Moreover, male roost fidelity is high for these palms and maximum group sizes are larger than those reported from roosts constructed from other plant species (Sagot et al. 2013). As clumped distributions of desirable roosts promote clumped distributions of males, females can potentially mate with any of a number of males in the surrounding area (Storz et al. 2000a,b; Gopukumar et al. 2005; Campbell et al. 2006). Finding unrelated males is not time‐consuming or energetically costly for females. Furthermore, it could be expected that energetic costs associated with seeking unrelated mates would be less than those associated with multiple generations of mating with close relatives. As females have access to and can mate with multiple unrelated males, inbreeding avoidance could be one of many criteria used in mate choice.

### Inbreeding in bat populations

Extent of inbreeding avoidance depends on its relative cost, compared to outbreeding (Waser et al. 1986; Kokko and Ots 2006; Olson et al. 2012). High costs of avoidance, such as delayed reproduction if unrelated males are not available, or decreased survival due to diseases, lead to inbreeding tolerance in natural populations (Pusey and Wolf 1996; Olson et al. 2012). On the other hand, excessive outbreeding is also detrimental in natural populations as it causes disruption of locally adapted gene complexes that are beneficial to adapt to immediate environments (Lynch 1991). In multiple studies, inbreeding has been associated with lowered offspring birthweight (Coltman et al. 1998). This is problematic for bats, because it increases nutritional dependency on mothers and makes it harder to thermoregulate (Kurta and Kunz 1987). *Uroderma bilobatum* appears not to be affected by high costs of inbreeding avoidance as it is common to find other social groups and/or solitary males in neighboring tents or palms (Timm and Lewis 1991; Lewis 1992). Moreover, it is expected that a benefit would be derived from reduced fidelity by acquiring beneficial alleles occurring in unrelated mates (Jennions and Petrie 2000; Di Battista et al. 2008), Thus, it is not surprising to find inbreeding avoidance mechanisms in this species.

The current data, in addition to previous findings regarding resource‐defense polygyny, suggest that habitat characteristics might influence patterns of inbreeding avoidance and female dispersal. Although only a few studies have reported inbreeding avoidance in other foliage roosting species (e.g., *Thyroptera tricolor –* Buchalski et al. 2014), similarities in their ecological requirements and social behavior suggest that inbreeding avoidance mechanisms might be widespread among *U. bilobatum*.

### Roosts and patterns of group genetic structure

Roosts are valuable resources for bats because they provide a space to carry out social interactions, but are relatively scarce (Kunz 1982; Kunz and Lumsden 2003) and costly to construct (Balasingh et al. 1995; Kalko et al. 2006; Rodríguez‐Herrera et al. 2007). Thus, it should not be surprising that roosts are one of the most important determinants of social systems in multiple bat species (Chaverri and Kunz 2010). However, to date, significance of roosts in social interactions has been overlooked and poorly understood (but see Sagot and Stevens 2012). Our findings suggest that in Peter's tent‐roosting bats, female group composition can be stable owing to a tendency of females to aggregate around suitable roosts, which is especially evident in roosts constructed from coconut palms. This palm is native to coastal areas (littoral zone) of South‐East Asia (Malaysia, Indonesia, Philippines) (Chan and Elevitch 2006; Baudouin and Lebrun 2009) and was probably introduced into West Africa and the Caribbean (including the Atlantic and Pacific coasts of Central America) by European explorers (Harries 1978) or Polynesians (Baudouin and Lebrun 2009). Currently, this palm is more abundant in human‐modified habitats, where people plant them as ornamentals. Due to the rather recent introduction in the continent, it is reasonable to assume that Peter's tent‐roosting bats historically roosted in native plants such as the palms *Attalea* spp. and *Cryosophila* spp. in forested areas. In the current study, Peter's tent‐roosting bats used these native palms infrequently, and social groups established in these plant species were inferred to be less structured than those found in coconut palms. Because coconut palms have similar leaf morphology, it allows Peter's tent‐roosting bats to construct tents of the same architecture. Thus, it seems that *U. bilobatum* has only relatively recently switched to use this non‐native palm in human‐modified habitats. Using these altered areas might reduce time and energy spent looking for plants to build new tents, as coconut palms are found in higher densities compared to native palms. Also, in human‐modified habitats, Peter's tent‐roosting bats may be released from predation pressure, particularly from monkeys and kites (Boinski and Timm 1985).

It appears that human introduction of the exotic coconut palm has influenced patterns social structure in *U. bilobatum,* by promoting social structuring and female natal dispersal.

## Data Accessibility

Capture location, microsatellite genotype, and population data will be deposited at Dryad. *Cyt‐b* haplotypes will be deposited at GenBank. Codes used to analyze the data have been properly cited in the text.

## Conflict of Interest

None declared.

## Supporting information


**Figure S1.** Haplotype network of sampled *U. bilobatum*. Colors represent different social groups. Each black line between black points indicates one point of mutation. Groups 1 2, 3, 11 and 12 are from Sarapiquí. Groups 4, 5, 6, 7, 8, 9 and 10 are from Carara.
