# Supplementary material for: Human‐modified habitats change patterns of population genetic structure and group relatedness in Peter's tent‐roosting bats
Source: Ecol Evol. 2016 Jul 29;6(17):6050–63. doi: 10.1002/ece3.2255 (PMC5016631; doi:10.1002/ece3.2255)
Supplement: Supplementary file 1 — Figure S1. Haplotype network of sampled U. bilobatum. Colors represent different social groups. Each black line between black points indicates one point of mutation. Groups 1 2, 3, 11 and 12 are from Sarapiquí. Groups 4, 5, 6, 7, 8, 9 and 10 are from Carara. [file ECE3-6-6050-s001.docx]

**SUPPLEMENTARY FIGURES**

**
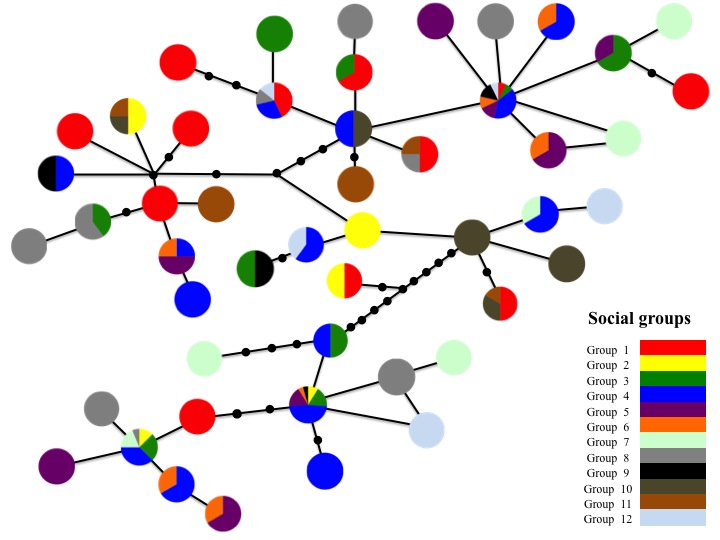
**

**Figure 1.** Haplotype network of sampled *U. bilobatum*. Colors represent different social groups. Each black line between black points indicates one point of mutation. Groups 1 2, 3, 11 and 12 are from Sarapiquí. Groups 4, 5, 6, 7, 8, 9 and 10 are from Carara.
